# Chemically mediated species recognition in two sympatric Grayling butterflies: *Hipparchia fagi* and *Hipparchia hermione* (Lepidoptera: Nymphalidae, Satyrinae)

**DOI:** 10.1371/journal.pone.0199997

**Published:** 2018-06-28

**Authors:** Manuela Pinzari, Marco Santonico, Giorgio Pennazza, Eugenio Martinelli, Rosamaria Capuano, Roberto Paolesse, Massimo Di Rao, Arnaldo D'Amico, Donatella Cesaroni, Valerio Sbordoni, Corrado Di Natale

**Affiliations:** 1 Department of Biology, University of Rome Tor Vergata, Rome, Italy; 2 Center for Integrated Research-CIR, Unit of Electronics for Sensor Systems, University Campus Bio-Medico, Rome, Italy; 3 Department of Electronic Engineering, University of Rome Tor Vergata, Rome, Italy; 4 Department of Chemical Science and Technology, University of Rome Tor Vergata, Rome, Italy; University of Arkansas, UNITED STATES

## Abstract

Pheromones are known to play an important role in butterfly courtship and may influence both individual reproductive success and reproductive isolation between species. Recent studies have focused on courtship in *Hipparchia* butterflies (Nymphalidae: Satyrinae) emphasizing morphological and behavioural traits, as well as genetic differences. Behavioural observations suggested a role for chemical cues in mate and species recognition, where the androconial scales on the forewings of these species may be involved in chemical communication between individuals. Cchemical-mediated signals have received relatively little attention in this genus. Here, we report the results of a three-year investigation of the volatile organic compounds (VOCs) released by *Hipparchia fagi* and *H*. *hermione* in order to identify differences in VOCs between these species where they live in syntopy. Our study was carried out using an array of cross-selective sensors known as an "Electronic Nose" (EN) that operates by converting chemical patterns into patterns of sensor signals. While the identity of volatile compounds remained unknown, sensor signals can be compared to identify similar or dissimilar chemical patterns. Based on the EN signals, our results showed that: 1) the two sexes have a similar VOCs pattern in *H*. *fagi*, while they significantly diverge in *H*. *hermione*; 2) VOCs patterns were different between females of the two species, while those of males were not.

## Introduction

Insects are well known for diverse systems of chemical communication [1][2][3][4]. They use chemicals for locating potential food sources [5], detecting predators [6] and recognizing kin [7]. Moreover, one of the most notable uses of volatile compounds in insects involves mate choice [8][9][10]. When seeking mates, chemical signals can convey a wide range of information on the identity and quality of prospective mates, and play a key role in species recognition, reproductive isolation and speciation [11][12][13]. In Lepidoptera, sexual communication usually involves multiple signals, including visual, tactile and olfactory cues to identify potential partners [8][14][15]. Pheromones of butterflies are multicomponent blends of chemicals that convey information about sex [16], age [17], mating status [18], and species [3][10]).

Lepidopteran pheromones are Volatile Organic Compounds (VOCs), the majority of which belong to families of esters, alcohols, ethers, and heterocyclic compounds [3][19][20]. Based upon the composition of the blend, pheromones facilitate male mate choice, and/or expelling other males from the mating area [21][22][23][24]. During nuptial flight and/or courtship, butterflies usually release pheromones when they are in close proximity to their partner, unlike some female moth species that release them to attract males from long distances [4]. The composition of pheromone blends is crucial for species recognition [4][14][21]. Chemical blends of closely related species often have the same major chemical component, but chemical messages of each species can differ in minor components or in the ratio between them [3].

Male butterflies secrete sex pheromones from modified wing scales, the androconia, or from small structures called "coremata" and "hair pencils", while females produce them in ductless glands in abdominal segments (or of legs or wings). In addition, butterflies produce cuticular lipids with low volatility that can act as male sex pheromones in some cases [25][26]. Several butterfly studies have described morphology and localization of scent organs [27][28][29][30], while others have analyzed the blend composition of scent pheromones to evaluate their roles in mate selection and species recognition [9][23][24] [31][32]. We hypothesize that olfactory communication between *Hipparchia* individuals likely occurs, as the presence of androconia on male forewings [33], courtship sequences with a ventilation phase (*Fanning*), and contact stimulation (*Bowing*) suggest [34][35][36]. Nevertheless, pheromones of this butterfly genus have not been analysed before.

Here we analysed the volatile compounds of two *Hipparchia* species: *H*. *fagi* (Scopoli, 1763), the woodland grayling, and *H*. *hermione* (Linnaeus, 1764), the rock grayling. These two closely related species are morphologically and genetically similar [37][38], and are sympatric and often syntopic, i.e., sharing similar habitats and flight periods. Even though *H*. *fagi* and *H*. *hermione* differ in courtship sequences which surely play important roles in finalizing mating rituals [36]), differences in sex pheromones may strengthen premating reproductive isolation mechanisms and facilitate species recognition.

Most previous studies of pheromones have used gas chromatography—mass spectroscopy (GC-MS) as this is the gold standard in analytical chemistry for VOCs analysis [3][8][9][23][26]. However, separation of blends into individual compounds strongly depends on the choice of the GC column material and on parameters used in the analysis. Thus, if a sample is made of a mixture of different and unknown compounds, this approach may not be able to detect them all, underestimating the original chemical complexity. In place of GC-MS analyses, we decided to use an array of partially selective gas sensors characterized by a broad sensitivity to a variety of compounds. This analysis, however, cannot separate and identify each relevant compound, but it can separate and compare the global pattern of volatile compounds between species.

The broad selectivity of our electronic sensors is comparable to that of animal olfactory receptors, where a limited number (i.e., about 300 of receptors in humans) are able to detect millions of unique odors, i.e., combinatorial selectivity [39] [40]. For this reason, these sensors are known as electronic noses (ENs). They transform VOCs into a pattern of sensor signals, where the identity of each volatile compound remains unknown, but the sensor signals can be compared to identify similar or dissimilar patterns of VOCs between species [41].

The main focus of our study was: i) to assess the production of volatile chemical patterns by males and females of both species, *Hipparchia fagi* and *H*. *hermione*, and ii) to compare the VOCs patterns in order to evaluate whether there were differences between sexes and species that might be involved in premating reproductive isolation mechanisms. We argue that analysis of the olfactory cues used by *H*. *fagi* and *H*. *hermione* males will help us better understand courtship interactions and of the importance of pheromones in mate recognition in butterflies.

## Materials and methods

No specific permissions were required for the study locations because these are not protected areas, and, therefore, the field studies did not involve endangered or protected species.

### Butterfly samples and study area

*Hipparchia fagi* and *H*. *hermione* used for chemical analyses were from Vallemare (Rieti, Italy, WGS84: 42.4836°N—13.1148°E), where the species are sympatric, their habitats overlap and adults fly together in summer. These species show very similar wing patterns, and identifying species can be difficult when using external features only, but the genital morphology provides good diagnostic characters for species identification [33][42][43]. Therefore, we first classified individuals based on their external features and confirmed species by examination of their genitalia. In the early stages of our study, we used reared individuals of *H*. *fagi* to finalize the procedures with the electronic nose (see section "Measurement protocol for butterflies"). When the protocol was optimized, we used individuals caught in the wild and performed laboratory experiments in the study area. These populations have been used in previous behavioural studies [36].

### Electronic nose

The VOCs of butterflies were measured with a gas sensor array (Electronic Nose) developed at the University of Rome Tor Vergata. The Electronic Nose (EN) was an ensemble of seven cross-selective quartz microbalance (QMB) gas sensors coated by seven different metalloporphyrins [44], based on 5,10,15,20-tetrakis-(4-butyloxyphenyl) porphyrins (buti-TPP) and characterized by the following metal ions: (Cu buti-TPP (sensor 1), Zn buti-TPP (sensor 2), Mn buti-TPP, (sensor 3), Fe buti-TPP (sensor 4), Sn buti-TPP (sensor 5), Ru buti-TPP (sensor 6) and Cr buti-TPP (sensor 7) [44].

Porphyrins are a versatile molecular framework to develop arrays of cross-selective sensors. The synthetic chemistry of porphyrins is well developed, since most of the periodic table elements have been incorporated into porphyrin rings, and peripheral porphyrin ring positions have been joined with several different functional groups [44].Porphyrins can interact with airborne molecules via different interaction mechanisms including coordination, van der Waals forces, hydrogen bonds, π- π and cation- π interactions. Furthermore, in the solid state the aggregation motif could also introduce additional sensing properties [44]. In spite of the large spectrum of mechanisms of interaction, these sensors can capture a large variety of volatile compounds, including alkanes, aromatics, amines, aldehydes, alcohols, and organic acids [45][46].

QMBs are piezoelectric resonators, and a change of the mass (Δm) on the quartz surface produces a change in the frequency (Δf) of the electric output signal of the oscillator circuit (S7 Fig) connected to the resonator [47]. The quantities Δm and Δf are linearly proportional in the low-perturbation regime. These QMBs have a fundamental frequency of 20 MHz, corresponding to a mass resolution of few nanograms. The sensor signal is the frequency of the output voltage.

The sensitivity of porphyrins coated QMB is rather variable, and it depends on the chemical characteristics of the volatile compound. The limit of detection is of the order of 100 ppb for diverse compounds such ethanol and ethyl acetate [48]. The interactions between volatile compounds and metalloporphyrins are reversible. In each measurement, the sensors of the EN were exposed for 2–3 mins to the mixture of volatile compounds to analyse, and then “cleaned” for 10–15 mins with a reference atmosphere, i.e., filtered ambient air.

### Measurement protocol for butterflies

In 2007, we tested the EN on *H*. *fagi* only (S1 Table) to assess its performance with butterfly VOCs and ascertain optimal environmental conditions for future experiments (see details of the pilot study in Supporting Information, S1 and S2 Figs). Both butterfly species (65 *H*. *fagi* and 63 *H*. *hermione*) were analyzed during three consecutive reproductive seasons: 14–20 July 2008, 13July-8August 2009; 13–26 July 2010 (Table 1). In 2008, we measured VOC emissions of males only, whereas in the following seasons, 2009 and 2010, we measured emissions of individuals of both sexes.

**Table 1:** Sample sizes of *Hipparchia fagi* and *H*. *hermione* in each season.

Species	Year	Conditions	Individuals	
			**Males**	**Females**
*H*. *fagi*	2007	In, out, sun/Perturbed, not perturbed	11	4
	2008	Out, not perturbed	21	
	2009	Out, not perturbed	10	9
	2010	Out, not perturbed	17	8
Total			59	21
*H*. *hermione*	2008	Out, not perturbed	23	
	2009	Out, not perturbed	4	5
	2010	Out, not perturbed	17	14
Total			44	19

Daily butterfly collections were carried out early in the morning, and all butterflies were kept in flight cages (50×50×50 cm) with food resources (over-ripe fruits and water), waiting to be tested. After 1–7 hours, each butterfly was transferred to a 314 ml glass jars for VOCs measurement. To prevent contamination of VOCs, each individual was manipulated as little as possible, using rubber gloves and tweezers, and glass jars were boiled in distilled water and dried before each experimental session. All measurements were taken between 09.00 a.m. and 7.00 p.m., in “not perturbed” conditions, within a temperature range of 19–30°C, with calm and sunny conditions and ambient photoperiod regime.

Once each butterfly was in a sealed glass jar, the extraction of VOCs started after 15–20 min, the time needed for a headspace composition to form and sent to the EN. Each butterfly was measured 3 to 5 times and, while all values were used in the initial pilot study, only individual means were used for statistical analyses. The headspace composition of an empty sealed glass jar was used as a control (reference air).

### Statistical analyses

The sensor response to a sample was calculated as the difference between the frequency of the sensor output voltage of the reference air and that of the air after sample exposure. The responses of the sensors were organized into a vector of 7 sensor-dimensional space. The group of responses to different samples were then ordered into matrices, whose rows represented the samples and columns the seven sensors. Signal differences between sexes and species and Principal Component Analysis (PCA) scores were evaluated with a parametric Kruskal-Wallis test, followed by Bonferroni correction for multiple comparisons.

PCA and Partial Least Squares Discriminant Analysis (PLS-DA) were used to explore and classify the Electronic Nose data, respectively [49][50]. The PLS-DA algorithm offers a simple and meaningful method to perform discriminant analysis. In PLS-DA, the class membership is represented as a vector, where the number of elements corresponds to the number of classes, and the class membership of data is expressed setting the corresponding vector element to one and all the others to zero. PLS-DA calculates a set of novel variables, called latent variables, to maximize the covariance with the class membership vectors. Like PCA, the latent variables can be plotted to provide a visual representation of the variation of the data. The performance of PLS-DA classifiers depends on the number of latent variables involved in the model. As PLS-DA classifiers tend to overfit the data, the choice of latent variables requires a cross-validation procedure where the prediction of the model is calculated on an independent set of data. We therefore used a Leave-one-out Cross Validation (LOOCV) procedure, where given n samples, the PLS-DA algorithm was trained n times on all data, except for one sample, and the statistics computed for that left-out sample. The average test-error rate over n trials was the estimated error rate. We used the cross-validated PLS-DA that is equivalent to a multivariate ANOVA [51].

The statistical significance of the PLS-DA classification models was evaluated with a permutation test, where the membership class was randomly attributed and a cross-validated PLS-DA model calculated each time [52]. For each permutation, a cross-validated PLS-DA model was calculated, and the Receiver Operating Characteristic (ROC) curve and the area under the ROC curve (AUROC) were evaluated as estimators of the statistical properties of the classification model [53]. The ROC curve is a typical tool to describe the performance of a binary classifier, and is made plotting the true positive rate versus the false positive rate with the threshold between the two group changes. The area under the curve (AUROC) yields the probability that a positive outcome is classified as negative [54].

PCA and PLS-DA were applied to the auto-scaled data matrix (S2, S3, S4 and S5 Tables), and the responses of each sensor were normalized to a zero mean and unitary variance. In some cases, the data were also linearly normalized to mitigate the dependence of the sensor signals on the VOCs concentration [55]. In linear normalization, the signal of each sensor is divided by the sum of the others. In case of an array of N linear sensors the response of the i-th sensor (Δ *fi*) to the i-th compound at concentration c (*cj*) (S7 Fig) is given by:

$$\Delta f_{i}^{*}=\Delta f_{i}/\sum_{j}\Delta f_{i}.$$

All data analyses were performed in Matlab R2011 (Mathworks, Natick, MA, USA).

## Results

Sensor 6 (Ru buti-TPP) detected significant differences between males and females of *H*. *hermione*, while sensor 2 (Zn buti-TPP) between males and females of *H*. *fagi*, sensor 5 (Sn buti-TPP) and sensor 4 (Fe buti-TPP) between females of *H*. *fagi* and *H*. *hermione* (Table 2). Details about the distribution of sensors responses are given in Supporting information (S3, S4, S5 and S6 Figs).

Since EN ability in detecting scents may exceed that of individual sensors [41], we assessed the differences between groups also on the whole sensor array data.

**Table 2:** P values for Kruskal-Wallis tests followed by Bonferroni correction. * p < 0.02, ** p < 0.01.

	Year	2009–2010	2009–2010	2008-2009-2010	*2009–2010*
	Sex	♂♂ vs. ♀♀		♂♂	♀♀
Sensor	Species	*H*. *fag*i	*H*. *hermione*	*H*. *fagi* vs. *H*. *hermion*e	
1: Cu buti-TPP		0.107	0.288	0.322	0.154
2: Zn buti-TPP		0.017*	0.351	0.283	0.184
3: Mn buti-TPP		0.087	0.124	0.897	0.114
4: Fe buti -TPP		0.055	0.192	0.051	0.011*
5: Sn buti-TPP		0.052	0.352	0.529	<0.000**
6: Ru buti -TPP		0.119	0.003**	0.413	0.084
7: Cr buti-TPP		0.046	0.206	0.025	0.808

### Intraspecific comparisons: Males *vs*. females

**Fig 1:**
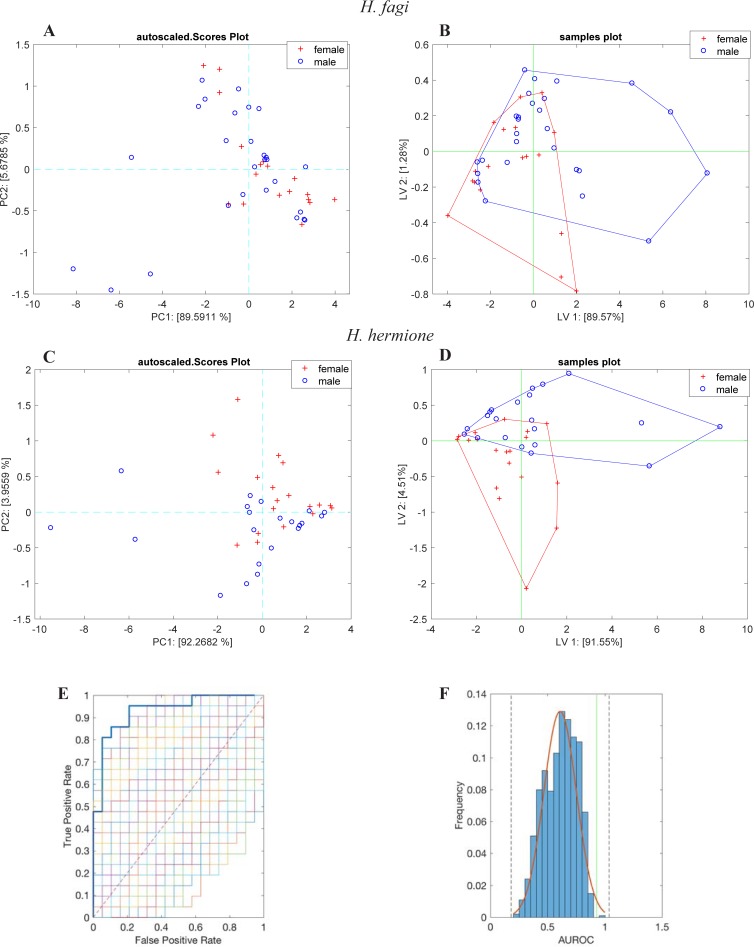
Sexes in comparison. PCA (A,C) and PLS-DA (B,D) displaying differences/similarities between males and females in *H*. *fagi* and *H*. *hermione*, respectively. The multivariate analyses were calculated using the whole data set (2008–2010). In *H*. *hermione*, PLS-DA model was validated by a random permutation test. Receiver Operating Characteristics Curves (ROC), obtained by each iteration (E), compare the classification with the hypothesis that data differs for sex respect to other random hypothesis. The frequency distribution of the AUROC (F) is fitted with a normal distribution; the vertical solid line is the AUROC obtained by the true class membership, and the dotted vertical lines indicate the 3σ limit of the distribution.

Both PCA and PLS-DA on sensor array data did not show any difference between sexes in *H*. *fagi* (Fig 1A and 1B). LOOCV, optimized with four latent variables, achieved only 68% of classification accuracy, showing that the discrimination between males and females in *H*. *fagi* was still rather poor, although sensor 2 (Zn buti-TPP) had a p-value close to 0.01,. Partial separation between the sexes in *H*. *hermione* was observed in the PCA (Fig 1C). PCA explained only 4% of the total variance due to PC2. While PLS-DA quantified the discrimination between sexes (Fig 1D). LOOCV produced a model with 3 latent variables. Due to our limited sample sizes, we could not carry out a proper training and test validation of the classifiers. Thus, the reliability of the PLS-DA model was tested with a permutation test (Fig 1E) with the ROC curves generated in each iteration of the permutation test. The comparison of the ROC curves indicated that classifier performed better than random class membership most of the time. This behaviour was evident when the frequency distribution of the area under the ROC (AUROC) was analysed (Fig 1F). The histogram was fitted with a normal distribution that quantified the probability of assigning a random class membership was more or less accurate than the separation of data into male and female groups. Specifically, in this species, the classification into males and females in *H*. *hermione* with 85% accuracy was achieved at a confidence level above 2σ.

### Interspecific comparison: *H*. *hermione vs*. *H*. *fagi*

**Fig 2:**
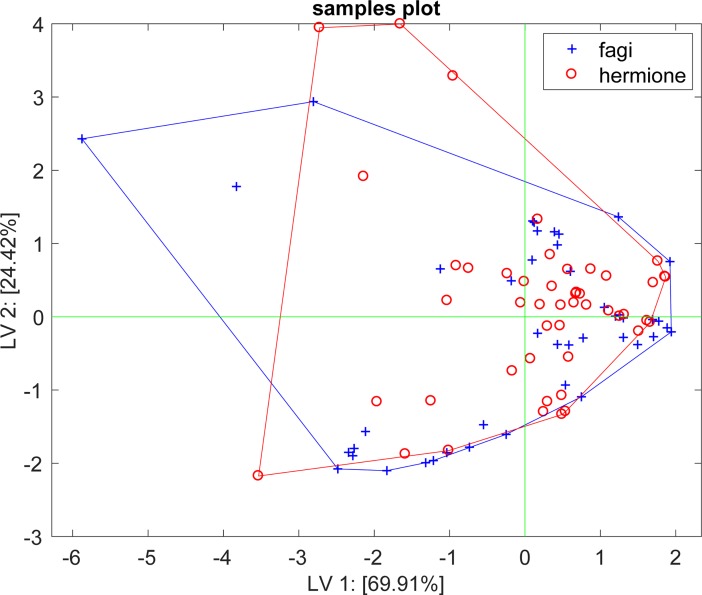
Males in comparison. PLS-DA displaying no appreciable differences between *H*. *fagi* and *H*. *hermione* in sensor responses to male VOCs. It was calculated on the whole data set (2008–2010). The plot of the first two latent variables showed an almost perfect overlap between the two groups.

Multivariate analysis of the sensor responses to the did not show any appreciable difference between *H*. *fagi* and *H*. *hermione* male volatile compounds. The application of PLS-DA to the whole data set did not improve the results: LOOCV resulted in 51% of accuracy similar to random choice. The plot of the first two latent variables of PLS-DA showed an almost perfect overlap between the two groups (Fig 2).

**Fig 3:**
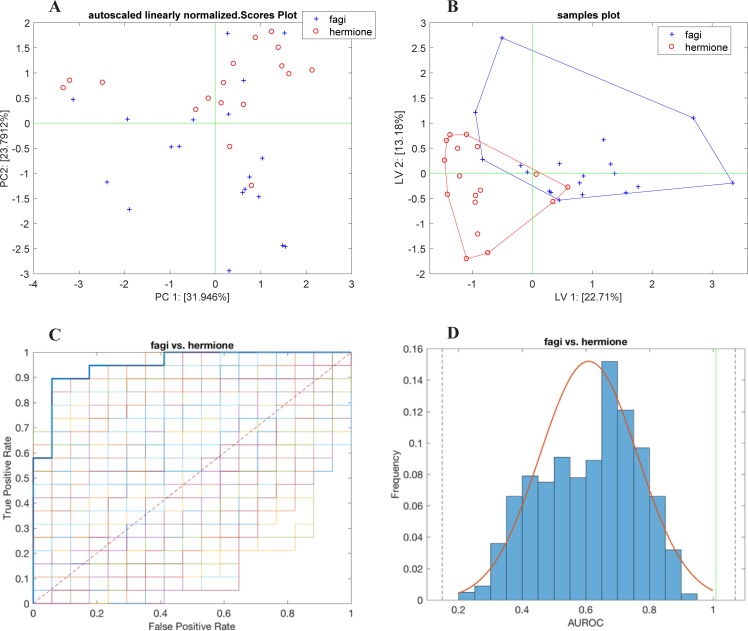
Females in comparison. PCA (A) and PLS-DA (B) separating sensor responses to female VOCs. The multivariate analysis were calculated using the whole data set (2009–2010). The PLS-DA model was validated by a random permutation test whose results are given as the collection of ROCs (C) and the AUROC distribution (D).

Separation between females of the two species is shown in Fig 3A. Quantitative description of the discrimination capabilities of the array was obtained by PLS-DA (Fig 3B). LOOCV produced a model with 4 latent variables and a cross-validated predicted accuracy of 85%. The collection of ROCs and the related AUROC distribution are shown in Fig 3C and 3D. Under the hypothesis of a normal distribution of the AUROC, the predicted accuracy (85%) was obtained with a confidence interval of 3σ.

## Discussion

The main purpose of this study was to assess the ability of EN to detect volatile organic compounds patterns produced by *Hipparchia* species and differences between sexes and/or species.We were able to convert *H*. *fagi* and *H*. *hermione* VOCs into seven gas sensor signals and EN revealed significant differences between *H*. *fagi* and *H*. *hermione* females, but not between males. When single species were considered, EN was able to distinguish between VOCs patterns released by the two sexes in *H*. *hermione*, but not in *H*. *fagi*.

### Cues used by males in individual recognition

Visual stimuli have a main role in prompting males to flight towards potential mates in several *Hipparchia* species, including *H*. *fagi* and *H*. *hermione*. They can, in fact, use visual stimuli as initial approach for species recognition and mate choice [34][56]. However, the flight approach is not always strictly selective [34][35][56] [57]. Males have a relatively poor ability in identifying conspecific partners from far away, but their ability improves as soon as the partner gets closer. Although *H*. *fagi* and *H*. *hermione* have a similar wing colour patterns, after the initial pursuit (*Flight* phase) interspecific interactions rarely continue. We hypothesize that species recognition occurs at the early stages of sexual interactions. Similarly, chemical and tactile stimuli most likely play an important role in courtship behaviour of these butterflies [34][56][57][58].

Our results show that VOCs patterns from females of *H*. *fagi* and *H*. *hermione* are significantly different as captured by the sensors 4 and 5 of the EN. Sensor 4 showed the largest response when exposed to VOCs released by *H*. *hermione*, and sensor 5 when it was exposed to VOCs from *H*. *fagi*. These responses suggest that the differences between species were not due to of concentration differences, but rather to a change in VOCs profile. Females producing different VOCs suggest that males may recognize conspecific partners on the basis of female smell at an early phase of the sexual interaction, i.e., nuptial flight. This is possible for *H*. *fagi*, as previous behavioral studies have shown that males identify conspecific females at an early stage of the sexual interaction [36]. Indeed, if male *H*. *fagi* accidentally chases a female *H*. *hermione*, he usually abandons the pursuit as soon as they are close to one other. On the contrary, VOCs are probably not the only cue used by *H*. *hermione* males to discriminate between conspecifics and *H*. *fagi* females. In fact, *H*. *hermione* males can undertake the nuptial flight with *H*. *fagi* females, and usually the courtship only ends because the female refuses the copulation attempt [36].

### Cues used by females in individual recognition

This study is consistent with previous observations on sexual behaviour [36] and suggests that female *H*. *fagi* and *H*. *hermione* do not recognize volatile olfactory cues for partner recognition, and instead use their olfactory system when they are in close contact with their partner during courtship. The mating behaviour of contact stimulation (*Bowing*), during which the male captures the female antennae between his forewings and ensures their physical contact with his androconia, develops differently in the two species. *H*. *fagi* females need longer *Bowing* and only a few repetitions of *Bowing* to accept to mate, while *H*. *hermione* females need more intense solicitation by short, highly repeated *Bowing*. These behavioural differences between species suggest that females place greater emphasis on tactile rather than chemical stimuli when accepting mates, and may therefore explain the lack of male VOCs differences in our study.

## Conclusions

The first use of an Electronic Nose to investigate VOCs in *Hipparchia* butterflies showed that courtship signaling involves an integrated sequence of signals, with at least two modes: sight and scent in addition to behavioral patterns that can involve contact-chemoreception signals, i.e., during *Bowing*. Volatile compounds are produced during sexual interactions of *H*. *fagi* and *H*. *hermione* suggesting an potential role of VOCs in partner recognition during the early stages of the interaction. To better understand the signals involved in species recognition, more manipulative behavioural experiments are needed, and studies on different species would also be useful for further comparisons.

The Electronic Nose proved to be sensitive enough to reveal significant differences between the patterns of volatile compounds of different sexes and species. This device does not provide direct information on the identity of the measured VOCs, rather it captures the global differences of the patterns. Further analyses are needed to identify the relevant compounds responsible for the chemical differences between species. We suggest that Electronic Noses provide unique opportunities to measure VOCs released by animal species in their natural environment because they can be operated in the field.

## Supporting information

S1 Text Pilot scheme. (DOCX)

S1 Table Sample size of *Hipparchia fagi* for each condition in the pilot scheme. (DOCX)

S2 Table 2007 measurement session: Pilot scheme. (DOCX)

S3 Table 2008 measurement session. (DOCX)

S4 Table 2009 measurement session. (DOCX)

S5 Table 2010 measurement session. (DOCX)

S1 Fig PCA displaying relative positions of males and females *H*. *fagi* pattern of VOCs to different conditions (IN, inside laboratory; OUT, environmental temperature; SUN, after basking; perturbed condition by provoking the flight of butterfly; not perturbed = motionless). Females were measured only when perturbed, all the other measurements were carried out on males. An empty glass jar was used as a control for the measurements. Repeated measures for each individual were included in PCA.
(TIF)

S2 Fig PCA displaying relative positions of males and females *H*. *fagi* pattern of VOCs to different conditions (IN, inside laboratory; OUT, environmental temperature; SUN, after basking; perturbed condition by provoking the flight of butterfly; not perturbed = motionless). It is the same plot in S1 Fig but the sensor responses are differently labeled for each butterfly.
(TIF)

S3 Fig Box plot of the distribution of each sensor signals of *H*. *fagi* females and males. P-values from the Kruskal-Wallis test are shown.
(EPS)

S4 Fig Box plot of the distribution of each sensor signals of *H*. *hermione* females and males. P-values from the Kruskal-Wallis test are shown.
(EPS)

S5 Fig Box plot of the distribution of each sensor signals of *H*. *fagi* and *H*. *hermione* males. P-values from the Kruskal-Wallis test are shown.
(EPS)

S6 Fig Box plot of the distribution of each sensor signals of *H*. *fagi* and *H*. *hermione* females. P-values from the Kruskal-Wallis test are shown.
(EPS)

S7 Fig Screenshot pc of libra nose software during a measurement session. The response of the i-th sensor (Δ fi) to the i-th compound at concentration c (cj) is given by: Δfi* ꞊ Δ fi / ∑j Δ fi.
(TIF)

## References

[1] Greenfield MD. Signalers and Receivers, Mechanisms and evolution of Arthropod communication Oxford University Press, 2002.

[2] Krieger J, Breer H. Olfactory Reception in Invertebrates. Science, 1999; 286 (5440): 720–723. 10531050 10.1126/science.286.5440.720

[3] Wyatt TD. Pheromones and animal behaviour: communication by smell and taste. Cambridge University Press, 2003.

[4] Matthews RW, Matthews JR. Chemical communication. In: Matthews RW, Matthews JR, editors. Insect Behavior, 2010. p.217-259.

[5] Pescador-Rubio A, Stanford-Camargo SG, Páez-Gerardo LE, Ramírez-Reyes AJ, Ibarra-Jiménez RA et al Trail marking by caterpillars of the silverspot butterfly dione juno huascuma. J Insect Sci., 2011;11:55 doi: 10.1673/031.011.5501 21861659 10.1673/031.011.5501 PMC3281466

[6] Sendoya SF, Freitas AVL, Oliveira PS. Egg‐laying butterflies distinguish predaceous ants by sight. Am Nat, 2009;174(1):134–140. doi: 10.1086/599302 19456265 10.1086/599302

[7] Leonhardt DS, Menzel F, Nehring V, Schmitt T. Ecology and Evolution of Communication in Social Insects. Cell, 2016; 164(6):1277–1287. doi: 10.1016/j.cell.2016.01.035 26967293 10.1016/j.cell.2016.01.035

[8] Costanzo K, Monteiro A. The use of chemical and visual cues in female choice in the butterfly *Bicyclus anynana*. Proc Biol Sci. 2007;274(1611): 845–851. doi: 10.1098/rspb.2006.3729 17251116 10.1098/rspb.2006.3729 PMC2093980

[9] Andersson J, Borg-Karlson A-K, Vongvanich N, Wiklund C. Male sex pheromone release and female mate choice in a butterfly. J Exp Biol. 2007; 210: 964–970. doi: 10.1242/jeb.02726 17337709 10.1242/jeb.02726

[10] Ring T, Cardé J, Millar G. Pheromones In: Resch VH, Cardé J, editors. Encyclopedia of Insects, Elsevier Academic Press, 2009; p.766–772.

[11] Phelan PL, Baker TC. Evolution of male pheromones in moths: reproductive isolation through sexual selection? Science, 1987; 235: 205–207. doi: 10.1126/science.235.4785.205 17778636 10.1126/science.235.4785.205

[12] Symonds MRE, Elgar MA. The evolution of pheromone diversity. Trends Ecol Evol, 2008;23: 220–228. doi: 10.1016/j.tree.2007.11.009 18308422 10.1016/j.tree.2007.11.009

[13] Smadja C, Butlin RK. On the scent of speciation: the chemosensory system and its role in premating isolation. Heredity 2009; 102: 77–97.Scott JA. Mating of Butterflies. J Res Lepid, 1973;11(2): 99–127. 10.1038/hdy.2008.55 18685572

[14] Li Chengzhe, Wang Hua, Chen Xiaoming, Yao Jun, Shi Lei, Zhou Chengli. Role of visual and olfactory cues in sex recognition in butterfly *Cethosia cyane cyane*. Scientific Reports, 2017; 7 (5033):1–9. 28694497 10.1038/s41598-017-04721-6 PMC5504021

[15] Grula JW, McChesney JD, Taylor OR. Aphrodisiac pheromones of the sulfur butterflies *Colias eurytheme* and *C*. *philodice* (Lepidoptera: Pieridae). J Chem Ecol, 1980; 6: 241–256.

[16] Nieberding CM, Fischer K, Saastamoinen M, Allen CE, Wallin EA, Hedenström E et al Cracking the olfactory code of a butterfly: the scent of ageing. Ecology Letters, 2012;15: 415–424. doi: 10.1111/j.1461-0248.2012.01748.x 22390373 10.1111/j.1461-0248.2012.01748.x

[17] Andersson J, Borg-Karlson A-K, Wiklund C. Sexual cooperation and conflict in butterflies: a male-transferred anti-aphrodisiac reduces harassment of recently mated females. Proc R Soc Lond B Biol Sci, 267; 1271–1275. 10.1098/rspb.2000.1138 PMC1690675 10972120

[18] Ryan MF. Insect chemoreception fundamental and applied. Kluwer Academic publishers New York, Boston, Dordtecht, London, Moscow, 2002.

[19] El-Sayed AM. The Pherobase: Database of Pheromones and Semiochemicals. 2017; Available from: http://www.pherobase.com

[20] Linn CE, Roelofs WL. Response specificity of male moths to multicomponent pheromones. Chem Senses, 1989;14: 421–427.

[21] Vane-Wright RI, Boppré M. Visual and chemical signalling in butterflies: functional and phylogenetic perspectives. Proc R Soc Lond B Biol Sci., 1993; 340 (1292): 197–205.

[22] Nieberding CM, de Vos H, Schneider MV, Lassance JM, Estramil N, Andersson J, et al The male sex pheromone of the butterfly Bicyclus anynana: towards an evolutionary analysis. PLoS One, 2008; 3(7):e2751 doi: 10.1371/journal.pone.0002751 18648495 10.1371/journal.pone.0002751 PMC2447158

[23] Darragh K, Vanjari S, Mann F, Gonzalez-Rojas MF, Morrison CR, Salazar C, Pardo-Diaz C, Merrill RM, McMillan WO, Schulz S, Jiggins CD. Male sex pheromone components in *Heliconius* butterflies released by the androconia affect female choice. PeerJ, 2017; 5:e3953 doi: 10.7717/peerj.3953 29134139 10.7717/peerj.3953 PMC5680698

[24] Heuskin S, Sevanderplanck M, Bacquet P, Holveck M, Kaltenpoth M, Engl T et al The composition of cuticular compounds indicates body parts, sex and age in the model butterfly *Bicyclus anynana* (Lepidoptera). Front Ecol Evol., 2014; 2(37): 1–16.

[25] Dapporto L. Cuticular mixture diversification in *L*. *megera* and *L*. *paramegaera*: the influence of species, sex, and population (Lepidoptera: Nymphalidae). Biol J Linn Soc Lond, 2007;91:703–710.

[26] Boppré M. Chemically mediated interactions between butterflies In: Vane-Wright RI, Ackery PR, editors. The biology of butterflies, Symposia of the Royal Entomological Society of London, Academic Press London; 1984; pp. 259–275.

[27] Ômura H, Itoh T, Wright DM, Pavulaan H, Schröder S. Morphological study of alar androconia in *Celastrina* butterflies. Entomol Sci, 2015;18: 353–359.

[28] Faynel C, Bálint Z. An overview of alar organs in French Guiana hairstreaks (Lepidoptera: Lycaenidae, Theclinae, Eumaeini) In: Diringer L, Faynel C, Bálint Z, editors. Lépidoptères de Guyane, Tome 5: Lycaenidae, 2012; pp. 46–54.

[29] Dion E, Monteiro A, Yew JY. Phenotypic plasticity in sex pheromone production in *Bicyclus anynana* butterflies. Sci Rep, 2016;6(39002):1–12. 27966579 10.1038/srep39002 PMC5155268

[30] Vanjari S, Mann F, Merrill RM, Schulz S, Jiggins CD. Male sex pheromone components in the butterfly *Heliconius melpomene*. Biorχivbeta, 2015 https://doi.org/10.1101/033506.

[31] Ômura H, Honda K. Chemical composition of volatile substances from adults of the Swallowtail *Papilio polytes* (Lepidoptera Papilionidae). Appl. Entomol. Zool. (Jpn.), 2005;40: 421–427.

[32] Kudrna O. A revision of the Genus *Hipparchia* Fabricius. E.W., Classey Ltd Editions, Faringdon, Oxon, England, 1977.

[33] Tinbergen N. Ethologische Beobachtungen am Samtfalter, *Satyrus semele* L. Journal für Ornithologie 1941;89: 132–144.

[34] Pinzari M (Manuela). A comparative analysis of mating recognition signals in Graylings: *Hipparchia statilinus* vs. *H*. *semele* (Lepidoptera: Nymphalidae, Satyrinae). Insect Behav, 2009;22 (3): 227–244.

[35] Pinzari M (Manuela), Sbordoni V. Species and mate recognition in two sympatric grayling butterflies: *Hipparchia fagi* and *H*. *hermione genava* (Lepidoptera). Ethol Ecol Evol, 2013;25(1): 28–51.

[36] Sbordoni V, Cesaroni D, Coutsis J, Bozano GC. Guide to the butterfly of the Paleartic region Satyrinae, Part V Omnes Artes, Milano, 2018.

[37] Dincă V, Montagud S, Talavera G, Hernández-Roldán J, Munguira ML, García-Barros E et al DNA barcode reference library for Iberian butterflies enables a continental-scale preview of potential cryptic diversity. Sci Rep. 2015; 5: 12395 doi: 10.1038/srep12395 26205828 10.1038/srep12395 PMC4513295

[38] Malnic B, Hirono J, Sato T, Buck L. Combinatorial receptor codes for odors. Cell, 1999; 96: 713–723. 10089886 10.1016/s0092-8674(00)80581-4

[39] Bushid C, Magnasco M, Vosshall L, Keller A. Humans can discriminate more than 1 trillion olfactory stimuli. Science, 2014; 343: 1370 doi: 10.1126/science.1249168 24653035 10.1126/science.1249168 PMC4483192

[40] Stitzel S, Arnecke M, Walt D. Artificial noses. Ann Rev Biomed Eng, 2011;13: 1–25. 21417721 10.1146/annurev-bioeng-071910-124633

[41] Higgins LG. The classification of European butterflies. Collins, 1975.

[42] Verity R. Le farfalle diurne d’Italia, Papilionida, vol. V Casa editrice Marzocco, Firenze, 1953.

[43] Paolesse R, Nardis S, Monti D, Stefanelli M, Di Natale C. Porphyrinoids for chemical sensor applications, Chem Rev, 2017;117: 2517–2583. doi: 10.1021/acs.chemrev.6b00361 28222604 10.1021/acs.chemrev.6b00361

[44] Di Natale C, Paolesse R, Macagnano A, Mantini A, Mari P, D’Amico A. Qualitative structure-sensitivity relationship in porphyrins based QMB chemical sensors. Sens Actuators B, 2000; 68:319–323.

[45] Ballantine D, White R, Martin A, Ricco A, Zellers E, Frye G, Wohltjen H. Acoustic Wave Sensors, Academic Press, (San Diego CA, USA), 1997.

[46] Catini A, Kumar R, Capuano R, Martinelli E, Paolesse R, Di Natale C. An exploration of the metal dependent selectivity of a metalloporphyrins coated quartz microbalance array. Sensors, 2016; 16: 1640–1652. 10.3390/s16101640 PMC5087428 27782032

[47] Santonico M, Pennazza G, Capuano R, Falconi C, Vink T, Knoble H et al Electronic noses calibration procedure in the context of a multicenter medical study. Sens. Actuators B, 2012; 173:555–561.

[48] Jolliffe I. Principal component analysis, Springer, New york, USA 2002.

[49] Barker M, Rayens W. Partial least squares for discrimination. J Chemom. 2003; 17: 166–173.

[50] Stahle L, Wold S. Multivariate analysis of variance (MANOVA). Chemometr Intell Lab 1990; 9: 127.

[51] Westerhuis J, Hoefsloot H, Smit S, Vis D, Smilde A, Van Velzen E et al Assessment of PLS-DA cross validation, Metabolomics, 2008;4: 81–89.

[52] Szymanska E, Saccenti E, Smilde A., Westerhuis J. Double check: validation of diagnostic statistics for PLS-DA models in metabolomics studies. Metabolomics, 2012;8: 3–16. doi: 10.1007/s11306-011-0330-3 22593721 10.1007/s11306-011-0330-3 PMC3337399

[53] Fawcett T. An introduction to ROC analysis, Pattern Recogn Lett, 2006; 27: 861–874.

[54] Horner G, Hierold C. Gas analysis by partial model building. Sens Actuators B Chem, 1990; 3: 173–184.

[55] Pinzari M (Manuela). Il comportamento riproduttivo dei lepidotteri: Neohipparchia statilinus ed altri satiridi. M.Sc. Thesis, University of Rome La Sapienza, 2004.

[56] Tinbergen N. The behaviour of the grayling butterfly In: Allen G, Unwin Ed Ltd, Editors. The Animal in Its World: Explorations of an Ethologist. Vol. I (Cambridge, MA: Harvard University Press), 1932–1972; p. 197–249.

[57] Pinzari M (Manuela). Corteggiamento e meccanismi di isolamento riproduttivo in due specie criptiche di farfalle del genere Hipparchia. Ph.d. thesis, University of Rome “Tor Vergata”, 2009 (available at: http://dspace.uniroma2.it/dspace/index.jsp)

[58] García-Barros E. Comparative data on the adult biology, ecology and behaviour of species belonging to the genera *Hipparchia*, *Chazara* and *Kanetisa* in central Spain (Nymphalidae: Satyrinae). Nota Lepidopterol 2000;23 (2): 119–140.

