# Supplementary material for: Chemically mediated species recognition in two sympatric Grayling butterflies: Hipparchia fagi and Hipparchia hermione (Lepidoptera: Nymphalidae, Satyrinae)
Source: PLoS One. 2018 Jun 28;13(6):e0199997. doi: 10.1371/journal.pone.0199997 (PMC6023170; doi:10.1371/journal.pone.0199997)
Supplement: S1 Text — (DOCX) [file pone.0199997.s001.docx]

**Chemically mediated species recognition in two sympatric Grayling butterflies: *Hipparchia fagi* and *Hipparchia hermione* (Lepidoptera: Nymphalidae, Satyrinae)**

Manuela Pinzari^1*^, Marco Santonico^3^, Giorgio Pennazza^3^, Eugenio Martinelli^2^, Rosa Maria Capuano ^2^, Roberto Paolesse ^4^, Massimo Di Rao^1^, Arnaldo D'Amico^2^, Donatella Cesaroni ^1^, Valerio Sbordoni ^1^, Corrado Di Natale ^2^

^1^ Department of Biology, University of Rome Tor Vergata, Rome, Italy

^2^ Department of Electronic Engineering, University of Rome Tor Vergata, Rome, Italy

^3^ Center for Integrated Research-CIR, Unit of Electronics for Sensor Systems, University Campus Bio-Medico, Rome, Italy.

^4^ Department of Chemical Science and Technology, University of Rome Tor Vergata, Rome, Italy

**Supporting information**

**S1 text. Pilot scheme**

We tested the Electronic Nose (EN) on fifteen reared individuals of *H. fagi* (S1 Table) to verify the applicability of this instrument to butterflies.

Each session started by carefully transferring a single living butterfly into a screw cap glass jar; then and the cap was sealed by parafilm. Caps were endowed with two gas-tight connectors to allow for the connection to the EN. One duct was used to refill the glass with filtered ambient air, and the other to convey the headspace from butterflies to the sensors of the EN.

Since temperature is an important factor that affects butterfly behaviour [1][2] and movement of wings could alter the release of chemical cues, measurements were performed in different settings to test the influence of temperature and movement. Besides, the physiological effects, the temperature also influences the volatility of the released compounds.

To test the influence of temperature, individuals were kept in three conditions until the measurement: 1) inside the laboratory at 24°C (IN), 2) outside the laboratory in the shade at the mean temperature of 29°C (OUT) and 3) outside the laboratory at 30°C after basking (SUN). Each butterfly was measured from two to four times.

To study the influence of wings motion, the individuals were kept in a PERTURBED or NOT PERTURBED condition at 30°C. The perturbation was simply obtained by shaking the butterfly container. Each individual was measured twice for each trial.

An empty glass jar was used as a control for the measurements.

| **Table S1 *–* Sample size of *Hipparchia fagi* for each setup in pilot scheme.** | | | | | |
| --- | --- | --- | --- | --- | --- |
| **Species** | **Year** | **Setup** | **Measures** | **Individuals** | |
|  |  |  |  | **Males** | **Females** |
| *H. fagi* | 2007 | IN, OUT, SUN* | 43 | 7 |  |
|  | 2007 | PERTURBED or NOT PERTURBED ** | 16 | 4 | 4 |
|  |  | Total | 59 | 11 | 4 |
| * in NOT PERTURBED conditions; ** in indoor conditions at 30°C. | | | | | |

**Results from pilot scheme**

The pilot experimentation provided useful information to set the protocol of measurement that we used in the following tests.

The PCA of sensors signals evidences the influence of temperature and wing movement on the sensors signals (S1 Fig). The arrangement of data along the first principal component (71% of explained variance) follows the temperature progression associated to the three experimental group (SUN, OUT, IN). The second principal component (13% of explained variance) carries the separation between perturbed and not perturbed individuals.

The influence of temperature and wing movement exceeds the differences among individuals and sexes. S2 Fig shows the same plot of S1 Fig but evidencing the response to same individuals.

Repeated measurements of same individuals in different conditions, and the test on males and females show that influence of temperature and wing movement exceeds the difference among sexes and individuals. PCA second principal component evidences a clear separation between the sensor responses to *H. fagi* males. Therefore, air temperature and wing movement of butterflies might play a role in the release of volatile compounds. As shown in S1 and S2 Figs , the dispersion of replicated measures does not affect the different experimental conditions.

**Conclusions**

The results of this pilot scheme showed that the measured sensor signals are only related to the VOCs emitted spontaneously by butterflies. Furthermore, this test showed that VOCs can be adequately measured without perturbing the animal and then limiting the study to the spontaneous VOCs emission.

Because of this test, we chose to measure individuals in “not perturbed” conditions to avoid unnecessary stresses in butterflies.

**References**

1. Van Dyck H, Matthysen E. Thermoregulatory differences between phenotypes in the speckled wood butterfly: hot perchers and cold patrollers? Oecologia, 1998;114: 326–334.
2. Pinzari M (Mario). Il comportamento territoriale di *Melitaea trivia* ([Denis & Schiffermüller],1775) (Lepidoptera, Nymphalidae). Bollettino dell’associazione romana di entomologia, 2000; 55 (1–4): 67–134.
