# Supplementary material for: Chemically mediated species recognition in two sympatric Grayling butterflies: Hipparchia fagi and Hipparchia hermione (Lepidoptera: Nymphalidae, Satyrinae)
Source: PLoS One. 2018 Jun 28;13(6):e0199997. doi: 10.1371/journal.pone.0199997 (PMC6023170; doi:10.1371/journal.pone.0199997)
Supplement: S1 Table — (DOCX) [file pone.0199997.s002.docx]

| **Table S1 *–* Sample size of *Hipparchia fagi* for each setup in pilot scheme.** | | | | | |
| --- | --- | --- | --- | --- | --- |
| **Species** | **Year** | **Setup** | **Measures** | **Individuals** | |
|  |  |  |  | **Males** | **Females** |
| *H. fagi* | 2007 | IN, OUT, SUN* | 43 | 7 |  |
|  | 2007 | PERTURBED or NOT PERTURBED ** | 16 | 4 | 4 |
|  |  | Total | 59 | 11 | 4 |
| * in NOT PERTURBED conditions; ** in indoor conditions at 30°C. | | | | | |
