# Supplementary material for: Chemically mediated species recognition in two sympatric Grayling butterflies: Hipparchia fagi and Hipparchia hermione (Lepidoptera: Nymphalidae, Satyrinae)
Source: PLoS One. 2018 Jun 28;13(6):e0199997. doi: 10.1371/journal.pone.0199997 (PMC6023170; doi:10.1371/journal.pone.0199997)
Supplement: S2 Table — (DOCX) [file pone.0199997.s003.docx]

**Table S2: 2007 measurement session: pilot scheme**

| **sensor 1 (Hz)** | **sensor 2 (Hz)** | **sensor 3 (Hz)** | **sensor 4 (Hz)** | **sensor 5 (Hz)** | **sensor 6 (Hz)** | **sensor 7 (Hz)** | **animal ID** | **measurement condition** |
| --- | --- | --- | --- | --- | --- | --- | --- | --- |
| 27.6 | 23.2 | 60.5 | 25.1 | 72.8 | 27.6 | 40.3 | 180 | IN, non perturbed |
| 10.9 | 16.9 | 39.1 | 20.3 | 40.2 | 20.2 | 27 | 180 | IN, non perturbed |
| 16.4 | 16.2 | 37.4 | 18.7 | 43.3 | 19.2 | 25.8 | 180 | IN, non perturbed |
| 32.1 | 24.2 | 78.3 | 32.1 | 204.4 | 38.8 | 47.5 | 180 | SUN |
| 30.5 | 23.4 | 73.3 | 31.1 | 207.6 | 35 | 41.7 | 180 | SUN |
| 32 | 22.7 | 66.5 | 31.4 | 212 | 33.8 | 46.3 | 180 | OUT |
| 29.1 | 23.2 | 67.4 | 31.4 | 205.8 | 35.7 | 47.1 | 180 | OUT |
| 28.7 | 21.7 | 61.6 | 27 | 137.8 | 30.6 | 49.2 | 180 | IN, non perturbed |
| 26.2 | 21.8 | 62.5 | 26.6 | 134.2 | 30.9 | 46.1 | 180 | IN, non perturbed |
| 15.9 | 18.8 | 45.2 | 20.5 | 55.5 | 20.9 | 31.8 | 185 | IN, non perturbed |
| 11.2 | 17.9 | 41.6 | 22.1 | 40.8 | 22.5 | 29.6 | 185 | IN, non perturbed |
| 15.7 | 26.2 | 60.6 | 28.5 | 65.3 | 28.2 | 44.1 | 185 | IN, non perturbed |
| 26.3 | 30.7 | 84.3 | 34.5 | 91.1 | 38.6 | 54.5 | 187 | IN, non perturbed |
| 18.9 | 15.9 | 34.3 | 18.1 | 34.6 | 19.1 | 24.4 | 187 | IN, non perturbed |
| 13.8 | 15.2 | 31.8 | 16.6 | 38.2 | 15 | 23.8 | 197 | IN, non perturbed |
| 23.9 | 23.2 | 55.2 | 25.5 | 60.1 | 26.1 | 41.4 | 197 | IN, non perturbed |
| 12 | 22.9 | 56.2 | 25.6 | 56.7 | 27.2 | 36.2 | 197 | IN, non perturbed |
| 40.5 | 56.9 | 205.7 | 35.3 | 177.5 | 47 | 58.7 | 197 | SUN |
| 33.6 | 41.6 | 208 | 31.2 | 159.2 | 43.5 | 53.5 | 197 | SUN |
| 30.7 | 35.4 | 229.4 | 31.8 | 122.4 | 46.3 | 54.5 | 197 | OUT |
| 28.1 | 30.5 | 213.8 | 31.2 | 105.6 | 48.5 | 53.3 | 197 | OUT |
| 25.7 | 25 | 146.1 | 28.7 | 110.5 | 43.5 | 48.4 | 197 | IN, non perturbed |
| 28.2 | 23.7 | 109.8 | 29.3 | 140.2 | 41.3 | 46.6 | 197 | IN, non perturbed |
| 31.3 | 26.6 | 72.8 | 30.6 | 78.3 | 31.9 | 48 | 198 | IN, non perturbed |
| 21.2 | 17.6 | 38.9 | 20.2 | 38.7 | 19.5 | 27.4 | 198 | IN, non perturbed |
| 36.2 | 25.9 | 107.2 | 34.8 | 213.7 | 47.6 | 50.4 | 198 | SUN |
| 31.3 | 26.8 | 91.6 | 34.3 | 220.3 | 42.4 | 52 | 198 | SUN |
| 39.1 | 25.3 | 83.3 | 32.4 | 217.3 | 38.1 | 50 | 198 | OUT |
| 36 | 23.4 | 75.9 | 31.4 | 219 | 35 | 47.1 | 198 | OUT |
| 26.8 | 22 | 63.9 | 27.3 | 154.6 | 30.4 | 47.6 | 198 | IN, non perturbed |
| 28.4 | 22.1 | 64.5 | 27.8 | 151.2 | 33 | 43.7 | 198 | IN, non perturbed |
| 17.3 | 11 | 25.2 | 13.6 | 29.6 | 12.7 | 18.7 | 203 | IN, non perturbed |
| 9.6 | 22.2 | 48.5 | 23.8 | 54.9 | 23 | 37.4 | 203 | IN, non perturbed |
| 26.5 | 25.4 | 59.7 | 27.8 | 68.4 | 28.3 | 44.3 | 203 | IN, non perturbed |
| 41.8 | 25.2 | 113.5 | 32.3 | 178.2 | 44.6 | 51.9 | 203 | SUN |
| 29.4 | 22.7 | 91 | 31 | 199.9 | 40 | 49.1 | 203 | SUN |
| 34.3 | 22.5 | 87.7 | 30.8 | 207.5 | 39.4 | 48.7 | 203 | OUT |
| 31.3 | 21.7 | 76.9 | 29.1 | 203.4 | 33.6 | 44.8 | 203 | OUT |
| 21.3 | 21.4 | 63.1 | 27.3 | 149 | 29.7 | 43.2 | 203 | IN, non perturbed |
| 24.3 | 19.9 | 57.8 | 25.4 | 135.9 | 27.4 | 41.1 | 203 | IN, non perturbed |
| 14.2 | 15.6 | 37.5 | 20.2 | 57.1 | 17.9 | 26.8 | 208 | IN, non perturbed |
| 20.1 | 17 | 41.7 | 19.1 | 53.6 | 21.5 | 31.9 | 208 | IN, non perturbed |
| 6.8 | 15.4 | 34.5 | 17.9 | 35 | 17.7 | 23.9 | 208 | IN, non perturbed |
| 3.8 | 19.4 | 63.9 | 23.8 | 111.1 | 25.1 | 43.6 | 210 | perturbed male |
| 7.9 | 21.2 | 73.8 | 26.2 | 163.2 | 29.6 | 42.9 | 210 | perturbed male |
| 6 | 23 | 85 | 29.4 | 193.9 | 34.5 | 46.3 | 310 | perturbed female |
| 3.7 | 20.1 | 67.6 | 27.5 | 198.5 | 29.9 | 40.9 | 310 | perturbed female |
| 10.4 | 26.5 | 88.7 | 34.2 | 196.5 | 38.7 | 54.4 | 211 | perturbed male |
| 13.3 | 24.1 | 77 | 37.2 | 195.1 | 33.3 | 43.2 | 211 | perturbed male |
| 12.7 | 21.5 | 70.4 | 29.9 | 152.1 | 30.7 | 44.6 | 311 | perturbed female |
| 6.3 | 24.9 | 79.6 | 33.4 | 159 | 35.4 | 50.5 | 311 | perturbed female |
| 5.7 | 0.5 | 5.7 | 0.8 | 2.7 | 1.6 | 1 | jar | control empty jar |
| 7.1 | 0.4 | 9.9 | 2.2 | 93.7 | 3.7 | 6.8 | jar | control empty jar |
| 5.4 | 18.5 | 65.6 | 24.8 | 143 | 26.5 | 39.3 | 212 | perturbed male |
| 6.4 | 20.9 | 71 | 28.5 | 149.1 | 28.9 | 41.9 | 212 | perturbed male |
| 3.9 | 24.6 | 92.5 | 32.1 | 181.6 | 36.8 | 51.6 | 312 | perturbed female |
| 5.3 | 28.6 | 108.4 | 36.5 | 234.9 | 42.5 | 58.2 | 312 | perturbed female |
| 4.1 | 28.1 | 102 | 36.5 | 213.6 | 41.8 | 57.7 | 213 | perturbed male |
| 12.7 | 25.5 | 91.7 | 36.4 | 268.9 | 38.4 | 52 | 213 | perturbed male |
| 3.2 | 25.8 | 86.3 | 35.6 | 175.1 | 37.2 | 52.9 | 313 | perturbed female |
| 7.1 | 27.7 | 98.9 | 36.1 | 176.5 | 41.3 | 60.6 | 313 | perturbed female |
