# Supplementary material for: Chemically mediated species recognition in two sympatric Grayling butterflies: Hipparchia fagi and Hipparchia hermione (Lepidoptera: Nymphalidae, Satyrinae)
Source: PLoS One. 2018 Jun 28;13(6):e0199997. doi: 10.1371/journal.pone.0199997 (PMC6023170; doi:10.1371/journal.pone.0199997)
Supplement: S3 Table — (DOCX) [file pone.0199997.s004.docx]

**Table S3: 2008 measurement session.**

| **sensor 1 (Hz)** | **sensor 2 (Hz)** | **sensor 3 (Hz)** | **sensor 4 (Hz)** | **sensor 5 (Hz)** | **sensor 6 (Hz)** | **sensor 7 (Hz)** | **sex** | **species** |
| --- | --- | --- | --- | --- | --- | --- | --- | --- |
| 119.27 | 28.3 | 142 | 28.93 | 59.3 | 62 | 55.9 | male | *H. hermione* |
| 145.5 | 32.97 | 159.17 | 36.57 | 74.17 | 59.47 | 65.9 | male | *H. hermione* |
| 119.17 | 27.3 | 132.93 | 29.7 | 60 | 43 | 52.23 | male | *H. hermione* |
| 107.43 | 14.77 | 54.5 | 36.23 | 40.73 | 24.53 | 35.23 | male | *H. hermione* |
| 146.17 | 19.97 | 68.9 | 46 | 50.13 | 49.13 | 44.23 | male | *H. hermione* |
| 183.9 | 40.1 | 172.35 | 52.55 | 96.7 | 45.1 | 78.05 | male | *H. hermione* |
| 95.7 | 22.53 | 114.9 | 32.77 | 41.37 | 18.07 | 34.47 | male | *H. hermione* |
| 208.97 | 31.9 | 165.13 | 59.4 | 88.47 | 76.87 | 67.1 | male | *H. hermione* |
| 49.43 | 12.93 | 37.93 | 18.93 | 26.07 | 2.87 | 19.93 | male | *H. hermione* |
| 41.5 | 11.1 | 29.17 | 16.53 | 22.53 | 3.5 | 17.27 | male | *H. hermione* |
| 174.63 | 38.8 | 158.3 | 51.1 | 95.27 | 72.8 | 71.67 | male | *H. hermione* |
| 47.17 | 14.1 | 26.13 | 19.43 | 26.97 | 3.57 | 22.37 | male | *H. hermione* |
| 43.07 | 12.57 | 23.5 | 17.7 | 24.17 | 4.6 | 19.67 | male | *H. hermione* |
| 126.13 | 22.83 | 116.67 | 39.5 | 55.97 | 26.63 | 41.23 | male | *H. hermione* |
| 61.6 | 15.4 | 38.77 | 19.7 | 33.93 | 10.4 | 26.33 | male | *H. hermione* |
| 102.1 | 25.47 | 64.13 | 28.1 | 56.93 | 18.47 | 44.93 | male | *H. hermione* |
| 115.57 | 27.2 | 138.73 | 28.47 | 57.03 | 61.47 | 53.33 | male | *H. hermione* |
| 94.53 | 23.63 | 61.9 | 28.23 | 50.9 | 15.1 | 41.53 | male | *H. hermione* |
| 230.83 | 33 | 174.4 | 60.1 | 92.9 | 74.53 | 55.28 | male | *H. hermione* |
| 224.5 | 31.73 | 170.13 | 65.57 | 88.73 | 74.93 | 59.47 | male | *H. hermione* |
| 130.5 | 23.77 | 130.87 | 41.27 | 50.97 | 20.77 | 40.37 | male | *H. hermione* |
| 62.4 | 14.55 | 73.08 | 21.58 | 25.78 | 18.48 | 20.28 | male | *H. hermione* |
| 74.37 | 19.13 | 69.93 | 16.9 | 38.43 | 23.77 | 35.4 | male | *H. hermione* |
| 67.5 | 16.5 | 75.67 | 19.03 | 34.27 | 29.23 | 32.4 | male | *H. fagi* |
| 72.8 | 18 | 82.87 | 20.5 | 36.53 | 32.63 | 34.97 | male | *H. fagi* |
| 157.78 | 27.43 | 130.45 | 55.95 | 66.3 | 77.65 | 53.73 | male | *H. fagi* |
| 77.47 | 12.97 | 36.4 | 30.9 | 30.27 | 30.4 | 28.73 | male | *H. fagi* |
| 63.02 | 16.14 | 87.88 | 24.26 | 30.16 | 6.58 | 28.2 | male | *H. fagi* |
| 62.57 | 15.53 | 85.37 | 23.93 | 29.63 | 6.57 | 28.73 | male | *H. fagi* |
| 72.97 | 12.03 | 34 | 30.23 | 30.6 | 29.47 | 28.4 | male | *H. fagi* |
| 84.27 | 13.83 | 40.53 | 36.47 | 36.9 | 29.37 | 35.17 | male | *H. fagi* |
| 55.6 | 9.2 | 27.65 | 24.83 | 23.2 | 17.25 | 22.55 | male | *H. fagi* |
| 105.03 | 17.8 | 49.3 | 44.3 | 43.48 | 32.8 | 41.03 | male | *H. fagi* |
| 190.7 | 30.77 | 147.83 | 65.47 | 79.77 | 109.77 | 65.4 | male | *H. fagi* |
| 53.63 | 9.77 | 27.33 | 23.6 | 21.9 | 14.3 | 20.6 | male | *H. fagi* |
| 79.07 | 19 | 93.27 | 27.87 | 36.37 | 15.63 | 29.43 | male | *H. fagi* |
| 101.5 | 25.9 | 55.37 | 27.3 | 59.87 | 35.6 | 45.07 | male | *H. fagi* |
| 114.53 | 26.1 | 78.77 | 33.57 | 60.53 | 26.37 | 46.33 | male | *H. fagi* |
| 108.07 | 28.03 | 60.37 | 28.9 | 63.67 | 37.1 | 50.37 | male | *H. fagi* |
| 294.2 | 38.47 | 189.57 | 57.83 | 102.77 | 137.67 | 78.1 | male | *H. fagi* |
| 195.63 | 27.77 | 148.93 | 50.63 | 69.63 | 44.1 | 52.47 | male | *H. fagi* |
| 91.93 | 22.27 | 68.67 | 31.03 | 46.17 | 6.9 | 38.53 | male | *H. fagi* |
| 125.17 | 29.03 | 94.67 | 41.03 | 63.17 | 9.47 | 52.23 | male | *H. fagi* |
| 112.1 | 27.43 | 130.7 | 26.83 | 56.93 | 50.8 | 53.4 | male | *H. fagi* |
