# Supplementary material for: Chemically mediated species recognition in two sympatric Grayling butterflies: Hipparchia fagi and Hipparchia hermione (Lepidoptera: Nymphalidae, Satyrinae)
Source: PLoS One. 2018 Jun 28;13(6):e0199997. doi: 10.1371/journal.pone.0199997 (PMC6023170; doi:10.1371/journal.pone.0199997)
Supplement: S4 Table — (DOCX) [file pone.0199997.s005.docx]

**Table S4: 2009 measurement session.**

| **sensor 1 (Hz)** | **sensor 2 (Hz)** | **sensor 3 (Hz)** | **sensor 4 (Hz)** | **sensor 5 (Hz)** | **sensor 6 (Hz)** | **sensor 7 (Hz)** | **sex** | **species** |
| --- | --- | --- | --- | --- | --- | --- | --- | --- |
| 11.47 | 3.67 | 6.6 | 6.8 | 10.07 | 6.33 | 3.2 | male | *H. fagi* |
| 56.4 | 21.33 | 32.13 | 40.48 | 30.98 | 20 | 32.6 | male | *H. fagi* |
| 69.33 | 24.3 | 35.5 | 46.83 | 36.67 | 29.03 | 36.33 | male | *H. fagi* |
| 109.77 | 36.03 | 69.07 | 60.83 | 58.6 | 50.57 | 55.07 | male | *H. fagi* |
| 84.23 | 27.3 | 49.33 | 44.47 | 47.5 | 37.67 | 40.6 | male | *H. fagi* |
| 53.8 | 16.6 | 32.35 | 29.6 | 31.43 | 23.5 | 30.15 | male | *H. fagi* |
| 39.83 | 13.3 | 22.1 | 25.78 | 21.03 | 16.08 | 23.55 | male | *H. fagi* |
| 38.33 | 12.4 | 17.93 | 25.07 | 20.3 | 15.9 | 21.6 | male | *H. fagi* |
| 48.5 | 15.2 | 14.4 | 33.23 | 25.4 | 23.57 | 25.23 | male | *H. fagi* |
| 41.5 | 12.77 | 19.5 | 28.2 | 21.53 | 16.5 | 20.27 | male | *H. fagi* |
| 49.3 | 15.33 | 14.8 | 29.8 | 25.23 | 20.4 | 22.77 | female | *H. fagi* |
| 78.17 | 29.07 | 72.97 | 53.1 | 50.67 | 31.07 | 43.93 | female | *H. fagi* |
| 227.87 | 60.37 | 113.57 | 116.13 | 132.17 | 165.73 | 105.35 | female | *H. fagi* |
| 44.5 | 14.67 | 30.13 | 14.7 | 31.7 | 17.27 | 18.7 | female | *H. fagi* |
| 79.83 | 22.93 | 40.3 | 44.7 | 41.63 | 35.6 | 37.87 | female | *H. fagi* |
| 167.5 | 40.23 | 105.23 | 70.77 | 85 | 87.27 | 68.67 | female | *H. fagi* |
| 55.15 | 18.53 | 19.75 | 27.53 | 26.6 | 22.03 | 24.68 | female | *H. fagi* |
| 98.58 | 32.53 | 78.03 | 53.88 | 54.45 | 40.6 | 49.98 | female | *H. fagi* |
| 45.03 | 15.6 | 15.07 | 28.73 | 23.7 | 20.2 | 25 | female | *H. fagi* |
| 57.93 | 14.47 | 18.13 | 42.07 | 28.43 | 22.93 | 29.8 | female | *H. fagi* |
| 17.58 | 5.53 | 13.25 | 7.45 | 12.23 | 8.25 | 8.43 | male | *H. hermione* |
| 44.18 | 9.85 | 26.8 | 15.28 | 32.48 | 20.2 | 18.25 | male | *H. hermione* |
| 36.8 | 12.37 | 13.43 | 21.87 | 18.8 | 14.63 | 13.73 | male | *H. hermione* |
| 22.53 | 5.4 | 5.6 | 6.33 | 13.77 | 8 | 8.23 | male | *H. hermione* |
| 46.6 | 15.4 | 14 | 33.2 | 24.47 | 20.4 | 23.67 | male | *H. hermione* |
| 34.58 | 6.93 | 17.03 | 12.53 | 21 | 16.8 | 14 | female | *H. hermione* |
| 97.07 | 28.13 | 22.57 | 54.1 | 50.17 | 41.5 | 47.33 | female | *H. hermione* |
| 52.73 | 15.17 | 9.77 | 33.17 | 26.27 | 22.4 | 25.27 | female | *H. hermione* |
| 23.86 | 6.82 | 16.2 | 9.68 | 23.8 | 13.64 | 9.8 | female | *H. hermione* |
