# Supplementary material for: Chemically mediated species recognition in two sympatric Grayling butterflies: Hipparchia fagi and Hipparchia hermione (Lepidoptera: Nymphalidae, Satyrinae)
Source: PLoS One. 2018 Jun 28;13(6):e0199997. doi: 10.1371/journal.pone.0199997 (PMC6023170; doi:10.1371/journal.pone.0199997)
Supplement: S5 Table — (DOCX) [file pone.0199997.s006.docx]

**Table S5: 2010 measurement session.**

| **sensor 1 (Hz)** | **sensor 2 (Hz)** | **sensor 3 (Hz)** | **sensor 4 (Hz)** | **sensor 5 (Hz)** | **sensor 6 (Hz)** | **sensor 7 (Hz)** | **sex** | **species** |
| --- | --- | --- | --- | --- | --- | --- | --- | --- |
| 136.23 | 39.53 | 52.87 | 57.6 | 79.4 | 63.13 | 74.3 | male | *H. fagi* |
| 134.37 | 35.37 | 46.23 | 51.2 | 64.37 | 55.6 | 60.27 | male | *H. fagi* |
| 89.7 | 27.47 | 53.03 | 40.87 | 53.23 | 51.23 | 48.33 | male | *H. fagi* |
| 86.2 | 29.23 | 52.3 | 41.23 | 57.2 | 58.43 | 50.97 | male | *H. fagi* |
| 86.07 | 29.3 | 51.27 | 40.33 | 58.77 | 32.03 | 49.47 | male | *H. fagi* |
| 239.07 | 28.13 | 98.3 | 78.4 | 92.1 | 59.8 | 76.17 | male | *H. fagi* |
| 192.87 | 28.3 | 86.2 | 74.83 | 80.5 | 51.73 | 67.3 | male | *H. fagi* |
| 201.63 | 25 | 92.8 | 67.55 | 79.78 | 57.8 | 67.48 | male | *H. fagi* |
| 306.63 | 78.13 | 80.1 | 153.83 | 131.4 | 128.13 | 148.5 | female | *H. fagi* |
| 404.5 | 86.67 | 98.07 | 161.7 | 160.97 | 147.5 | 165.83 | female | *H. fagi* |
| 266.17 | 67 | 64.17 | 125.47 | 119.97 | 86.87 | 128.27 | female | *H. fagi* |
| 96.9 | 31.93 | 55.47 | 50.67 | 55.17 | 37.73 | 57.53 | female | *H. fagi* |
| 98.37 | 30.87 | 64.93 | 51.1 | 57.3 | 41.23 | 58.17 | female | *H. fagi* |
| 82.53 | 27.33 | 54.47 | 42.07 | 49.23 | 37.37 | 49.17 | female | *H. fagi* |
| 78.83 | 27.43 | 52.53 | 39 | 47.37 | 38.33 | 47.6 | female | *H. fagi* |
| 80.43 | 27.83 | 53.03 | 38.93 | 51 | 40.37 | 48.3 | female | *H. fagi* |
| 179.7 | 41.8 | 71.23 | 57.27 | 79.83 | 35.57 | 69.13 | female | *H. fagi* |
| 132.03 | 37.73 | 49.2 | 46.6 | 68.67 | 47.33 | 63.2 | female | *H. fagi* |
| 77.77 | 27.03 | 53.73 | 34.63 | 48.13 | 45.57 | 47.03 | female | *H. fagi* |
| 162.3 | 39.9 | 50.7 | 67.3 | 78.07 | 56.73 | 64.73 | female | *H. fagi* |
| 79.07 | 28.23 | 43.03 | 42.63 | 48.67 | 47.67 | 43.97 | female | *H. fagi* |
| 221.83 | 37.43 | 89.43 | 83.17 | 102.2 | 56.4 | 81.37 | female | *H. fagi* |
| 217.6 | 36.23 | 88.87 | 84.47 | 96.7 | 42.17 | 78.87 | female | *H. fagi* |
| 142.97 | 30.8 | 77.1 | 69.13 | 67.87 | 35.47 | 64.93 | female | *H. fagi* |
| 128.13 | 31.4 | 84.5 | 64.2 | 66.27 | 33.63 | 57.37 | female | *H. fagi* |
| 133.2 | 38.97 | 55.27 | 60.7 | 68.2 | 35.2 | 69.57 | male | *H. hermione* |
| 76.77 | 26.23 | 37.27 | 40.77 | 44.97 | 44.27 | 42.2 | male | *H. hermione* |
| 222.77 | 33.93 | 61.5 | 65.83 | 91.8 | 71.77 | 69.83 | male | *H. hermione* |
| 168.8 | 35.13 | 31.2 | 53.3 | 77.2 | 61.57 | 58.63 | male | *H. hermione* |
| 157.23 | 37.47 | 33.33 | 48.2 | 74.1 | 58.2 | 64.37 | male | *H. hermione* |
| 89.77 | 28.63 | 45.4 | 40.17 | 60.03 | 34.63 | 51.03 | male | *H. hermione* |
| 70.03 | 24.13 | 45.87 | 33.1 | 47.47 | 26.67 | 40.43 | male | *H. hermione* |
| 100.33 | 31.67 | 37.53 | 41.5 | 64.63 | 39.8 | 54.3 | male | *H. hermione* |
| 90.27 | 31.87 | 54.07 | 43.6 | 64.13 | 31.57 | 52.07 | male | *H. hermione* |
| 215.07 | 49.43 | 48.67 | 77.9 | 109.4 | 6.83 | 90.53 | male | *H. hermione* |
| 81.23 | 28.07 | 48.83 | 39.6 | 57.73 | 14.6 | 47.73 | male | *H. hermione* |
| 90.27 | 30.13 | 48.9 | 45.33 | 62.8 | 13.27 | 51.53 | male | *H. hermione* |
| 264 | 61.3 | 86.57 | 94.77 | 93.8 | 40.2 | 95.53 | male | *H. hermione* |
| 205.27 | 56.03 | 84.97 | 92.33 | 93.63 | 55 | 91.8 | male | *H. hermione* |
| 414.2 | 79.2 | 119.2 | 153.1 | 127.8 | 128.57 | 162.6 | female | *H. hermione* |
| 435.07 | 76.07 | 180 | 171.4 | 134.37 | 105.47 | 171.63 | female | *H. hermione* |
| 557.3 | 84.63 | 219.5 | 219.5 | 209.57 | 163.8 | 226.1 | female | *H. hermione* |
| 132.73 | 41.73 | 62.27 | 67.93 | 69.77 | 48.67 | 70.7 | female | *H. hermione* |
| 129.07 | 41.23 | 61.1 | 71.07 | 77.9 | 54.77 | 70.47 | female | *H. hermione* |
| 111.97 | 36.43 | 55.6 | 59.67 | 64 | 45 | 61.17 | female | *H. hermione* |
| 116.2 | 36.97 | 61.67 | 53.13 | 72.03 | 60.8 | 68.03 | female | *H. hermione* |
| 62.23 | 21.6 | 42.8 | 29.13 | 37.97 | 37.1 | 36.8 | female | *H. hermione* |
| 50.27 | 18.07 | 38.93 | 25.83 | 30.23 | 35 | 28.87 | female | *H. hermione* |
| 52.67 | 18.17 | 40.07 | 25.87 | 32.33 | 36.8 | 29.7 | female | *H. hermione* |
| 47.9 | 18.23 | 37 | 22.3 | 29.77 | 32.83 | 27.23 | female | *H. hermione* |
| 243.1 | 37.23 | 75.8 | 64.1 | 106.1 | 103.2 | 78.6 | female | *H. hermione* |
| 159.17 | 39.6 | 40.27 | 51.83 | 80.57 | 87.1 | 65.43 | female | *H. hermione* |
| 123.17 | 36.8 | 38.07 | 46.07 | 72.5 | 78.03 | 59.17 | female | *H. hermione* |
| 110.77 | 35.83 | 40.5 | 48.07 | 70.97 | 72.67 | 58.43 | female | *H. hermione* |
| 89.9 | 29.47 | 45.77 | 40.6 | 59.43 | 59.3 | 50.2 | female | *H. hermione* |
| 143.87 | 31.57 | 78.97 | 68.47 | 72.2 | 54.67 | 64.27 | female | *H. hermione* |
