# Supplementary figures and images for: Chemically mediated species recognition in two sympatric Grayling butterflies: Hipparchia fagi and Hipparchia hermione (Lepidoptera: Nymphalidae, Satyrinae)
Source: PLoS One. 2018 Jun 28;13(6):e0199997. doi: 10.1371/journal.pone.0199997 (PMC6023170; doi:10.1371/journal.pone.0199997)

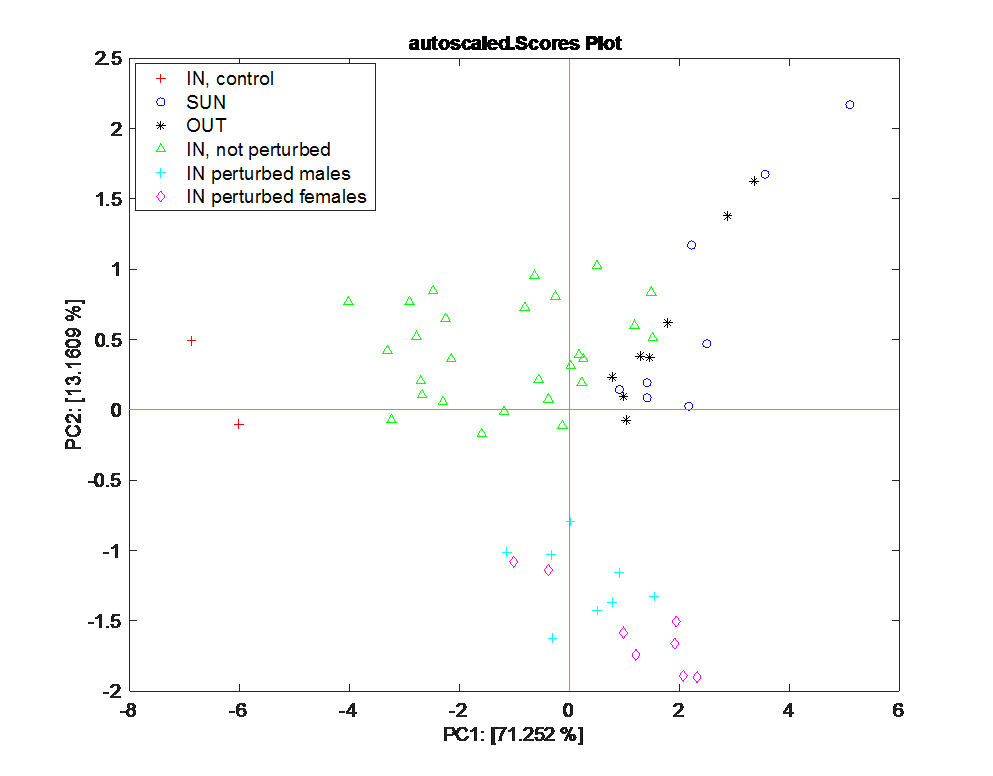

Supplement: S1 Fig — Females were measured only when perturbed, all the other measurements were carried out on males. An empty glass jar was used as a control for the measurements. Repeated measures for each individual were included in PCA. (TIF) [file pone.0199997.s007.tif]

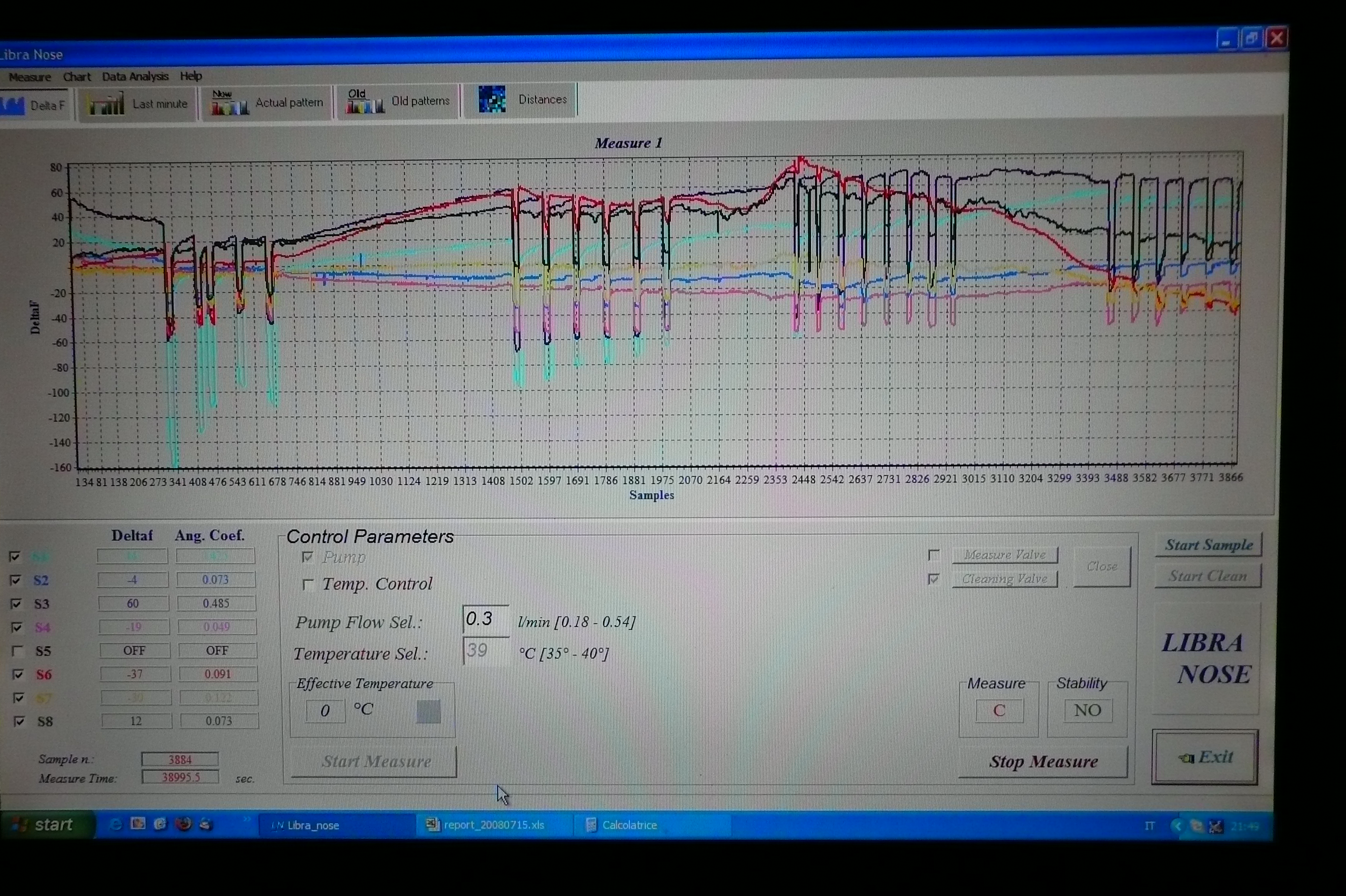

Supplement: S2 Fig — It is the same plot in S1 Fig but the sensor responses are differently labeled for each butterfly. (TIF) [file pone.0199997.s008.tif]

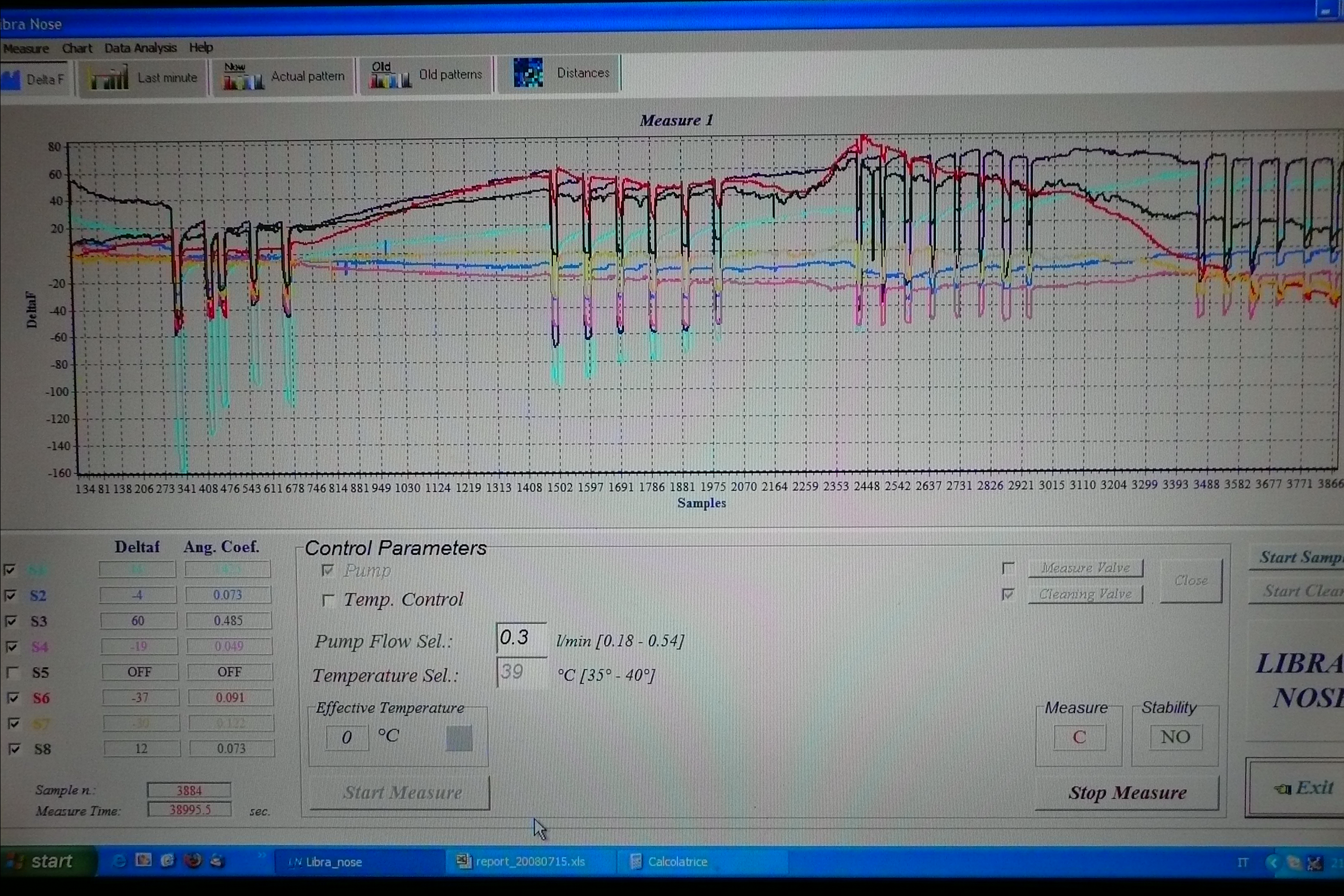

Supplement: S7 Fig — The response of the i-th sensor (Δ fi) to the i-th compound at concentration c (cj) is given by: Δfi* ꞊ Δ fi / ∑j Δ fi. (TIF) [file pone.0199997.s013.tif]
